# Bioactive Peptides from Lupin (*Lupinus angustifolius*) Prevent the Early Stages of Atherosclerosis in Western Diet-Fed ApoE^–/–^ Mice

**DOI:** 10.1021/acs.jafc.2c00809

**Published:** 2022-06-29

**Authors:** Guillermo Santos-Sánchez, Ivan Cruz-Chamorro, Ana Isabel Álvarez-Ríos, Nuria Álvarez-Sánchez, Beatriz Rodríguez-Ortiz, Ana Isabel Álvarez-López, María-Soledad Fernández-Pachón, Justo Pedroche, Francisco Millán, María del Carmen Millán-Linares, Patricia Judith Lardone, Ignacio Bejarano, Antonio Carrillo-Vico

**Affiliations:** † Instituto de Biomedicina de Sevilla, IBiS (Universidad de Sevilla, HUVR, Junta de Andalucía, CSIC), 41013 Seville, Spain; ‡ Departamento de Bioquímica Médica y Biología Molecular e Inmunología, Universidad de Sevilla, 41009 Seville, Spain; § Departamento de Bioquímica Clínica, Unidad de Gestión de Laboratorios, Hospital Universitario Virgen del Rocío, 41013 Seville, Spain; ∥ Área de Nutrición y Bromatología, Departamento de Biología Molecular e Ingeniería Bioquímica, Universidad Pablo de Olavide, Ctra Utrera Km 1, 41013 Seville, Spain; ⊥ Department of Food & Health, Instituto de la grasa, CSIC, Ctra Utrera Km 1, 41013 Seville, Spain

**Keywords:** Alcalase, bioactive peptides, cholesterol, inflammation, lupin, oxidative stress, atherosclerosis

## Abstract

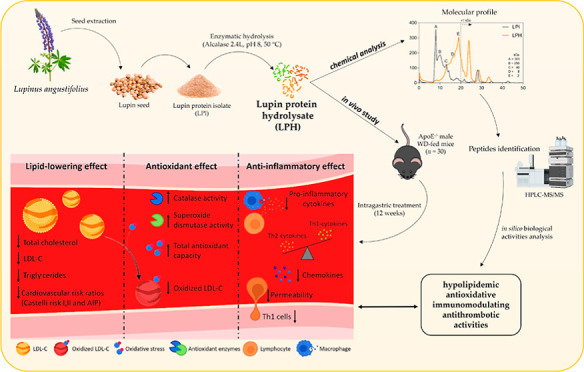

We have previously reported the *in vitro* hypocholesterolemic, anti-inflammatory, and antioxidant effects of Alcalase-generated lupin protein hydrolysate (LPH). Given that lipoprotein deposition, oxidative stress, and inflammation are the main components of atherogenesis, we characterized the LPH composition, in silico identified LPH–peptides with activities related to atherosclerosis, and evaluated the *in vivo* LPH effects on atherosclerosis risk factors in a mouse model of atherosclerosis. After 15 min of Alcalase hydrolysis, peptides smaller than 8 kDa were obtained, and 259 peptides out of 278 peptides found showed biological activities related to atherosclerosis risk factors. Furthermore, LPH administration for 12 weeks reduced the plasma lipids, as well as the cardiovascular and atherogenic risk indexes. LPH also increased the total antioxidant capacity, decreased endothelial permeability, inflammatory response, and atherogenic markers. Therefore, this study describes for the first time that LPH prevents the early stages of atherosclerosis.

## Introduction

Atherosclerosis lesions begin with endothelial dysfunction that causes abnormal transport of plasmatic low-density lipoprotein cholesterol (LDL-C) into the subendothelial space where they are oxidized, generating oxidized LDL (oxLDL). In this phase, oxLDL promotes an inflammatory response in the arterial endothelium.^1^ Consequently, endothelial permeability increases leukocyte recruitment (lymphocytes and macrophages) to the subendothelial space, which produce pro-inflammatory cytokines.^1−3^ After massive uptake of oxLDL, macrophages give rise to foam cells. All these previous steps to the atheroma plaque consolidation are widely described in the literature as the early stage of atherosclerosis,^4−8^ which play a key role in the further progression to late atherosclerosis, in which the foam cells participate in the fatty streak formation.^9^ High plasmatic total cholesterol (TC), triglycerides (TG), and LDL-C levels, as well as a low plasmatic level of high-density cholesterol (HDL-C) are identified as the main risk factors for cardiovascular diseases,^10^ pointing out that the diet develops a central role in the prevention of atherosclerosis.^11^

Dyslipidemia is associated with a high production of radical oxygen species. In fact, oxidative stress plays a pivotal role in atherosclerotic disease by participating in endothelial dysfunction, lipid oxidation, leukocyte migration, and inflammation.^12^

Recent studies have suggested that proteins and peptides from food, in addition to maintaining their nutritional value, exert biological activities such as antioxidant, antihypertensive, hypocholesterolemic, or immunomodulatory activities.^13^ In this regard, the protective activity against atherosclerosis progression and the hypocholesterolemic effect of a protein from *Lupinus albus*has been previously described in rabbits through a nutritional approach.^14,15^ In addition, *in vitro* approaches have shown that hydrolysates of*L. albus*obtained by pepsin or trypsin interfere with the cholesterol metabolism pathway in HepG2 cells.^16,17^

These studies were the basis for the opening of a new research field based on the study of lupin protein hydrolysates and peptides as new nutraceuticals. In this line, our group has previously shown the *in vitro* antioxidant and anti-inflammatory actions of*Lupinus angustifolius*protein hydrolysate (LPH) generated by the food grade-enzyme Alcalase.^18−20^ We have also described that the LPH treatment reduces abdominal adiposity and improves fatty liver disease, reducing the steatosis, inflammation, and increasing the antioxidant status in Western diet (WD)-fed mice.^21^ Furthermore, the molecular analysis of the *in vivo* LPH effects on the cholesterol metabolic pathway of liver showed that LPH regulates the activation of HMGCoAR, the main enzyme responsible for cholesterol synthesis, and the protein levels of LDL-C receptor (LDL-R).^22^ In addition, we have reported the beneficial effects of a functional beverage based on LPH on the antioxidant and anti-inflammatory status as well as decreasing of the atherogenic index in healthy subjects.^23^

In light of these considerations and because no previous studies have addressed the pleiotropic actions of bioactive peptides from lupin in the key components of the early stages of atherosclerosis (hyperlipidemia, oxidative stress, and inflammation), the main objective of this study was to analyze the effects of LPH on early risk factors for atherosclerosis in a WD-induced hypercholesterolemia model in ApoE^–/–^ mice. ApoE, a glycoprotein component of lipoproteins, plays a central role in fat metabolism by binding to LDL-R. Thus, ApoE absence predisposes to hypercholesterolemia and atherosclerosis,^24^ reason why ApoE knockout mice are widely used in studies related to dyslipidemia.

In the present work, we first characterized the LPH composition and the number of sequences with biological activities related to atherosclerosis risk factors. Subsequently, we studied the LPH treatment effects on the blood lipid levels and oxidative stress status, as well as on the aortic inflammation, and the level of atherogenic markers in WD-fed ApoE^–/–^ mice.

## Materials and Methods

### Lupin Protein Hydrolysate Preparation and Identification of Peptides

LPH was prepared following the previously described protocol.^20^ Briefly, lupin protein isolate (LPI) was hydrolyzed in a bioreactor using the followed conditions: 15 min with Alcalase 2.4L enzyme (2.4 AU/g; Novozymes, Bagsvaerd, Denmark) at pH = 8 (maintained throughout the process by adding 1 N NaOH), temperature of 50 °C, and E/S = 0.3 AU/g protein. For Alcalase inactivation, the temperature was increased up to 85 °C for 15 min and the subsequent centrifugation (10,437*g* for 15 min) allowed for the collection of the supernatant (=LPH). The latter was lyophilized and stored at 20–25 °C.^20^

Concentration of proteins,^20^ total dietary fiber,^25^ oil content,^26^ soluble sugars,^27^ polyphenols,^28^ degree of hydrolysis,^29^ molecular weights profile,^20^ and peptides identification^22^ were measured as previously described (see the Supporting Information for more detail).

Peptides identification allowed us to know the exact amino acid distribution and the length of each sequence.

The protein sequence database of*L. angustifolius*(31,386 sequences) was downloaded from UnitProt and used for the identification of raw data spectra using Proteome Discoverer v1.3 (Thermo) with the Mascot search engine v2.3.02. To identify the sequences with demonstrated bioactive motifs, the peptides were analyzed using the BIOPEP-UWM database.^30^

### Animals and Experimental Design

ApoE^–/–^ mice were housed in a colony at the Instituto de Biomedicina de Sevilla (IBiS) Animal Facility with a 12 h light/dark schedule (lights on at 8:00 a.m.) and *ad libitum* access to food and water. WD (45% calories from fat) was from the Special Diet Production Section of the University of Granada (Granada, Spain). The diet ingredients and nutritional profile are described in Supporting Information, Table S1. 6 week-old male mice (*n* = 60) were randomly divided into two groups and intragastrically treated with 100 mg/kg LPH (LPH group, *n* = 30) or vehicle (control group, Ctrl, *n* = 30) for 12 weeks, 5 days/week. Five independent experiments with 5–7 mice per group were carried out in this study. Mice were given WD with a standard cholesterol concentration for 12 weeks, representing an early stage of atherosclerosis consisting of hyperlipidemia, oxidative stress, and aorta inflammation according to previous studies.^7,31,32^

The selected LPH dose was based on previous internal tests. The equivalent human dose was calculated to be 8.12 mg/kg, according to Reagan-Shaw et al.^33^ Lyophilized LPH was dissolved in a physiological saline solution containing 0.25% carboxymethylcellulose (Sigma-Aldrich, St. Louis, MO, USA). Thirty mice were included in each experimental group according to the sample size calculated using the nQuery sample size software (Statsols, San Diego, CA, USA). Individual body weight and food intake were measured and recorded weekly. 12 h fasted mice were sacrificed by an intraperitoneal injection of sodium thiopental (50 mg/kg, B. Braun Medical SA, Barcelona, Spain). Blood was collected in MiniCollect tubes EDTA (Greiner Bio-One, Kremsmünster, Austria) by cardiac puncture and then animals were perfused with phosphate buffered saline (PBS) for 5 min using the FH100 peristaltic pump (Thermo Scientific, Vantaa, Finland). The aorta arteries were collected and stored at −80 °C until use. Plasma was separated by centrifugation (3000*g*, 4 °C, 10 min) and stored at −20 °C until use. All experimental procedures were approved by the ethics committee of the University Hospital Virgen Macarena-Virgen del Rocío and the Andalusian Ministry of Agriculture, Fisheries and Development (reference number 21/06/2016/105) and were carried out under Spanish legislation and the EU Directive 2010/63/EU for animal experiments.

### Blood–Lipid Profile

TC, HDL-C, LDL-C, and TG levels were quantified in plasma through chemiluminescence immunoassay techniques using the COBAS E 601 modular analyzer (Roche Diagnostic, Bassel, Switzerland). Lipoprotein risk ratios, including Castelli risk index (CRI) (I) (TC/HDL-C), CRI (II) (LDL-C/HDL-C), and atherogenic index of plasma (AIP) [Log_10_(TG/HDL-C)], were calculated. The sensitivity and inter/intra assay precision of the assays are shown in Supporting Information, Table S2.

### Antioxidant Capacity

Total antioxidant capacity (TAC) (Cell Biolabs, CA, USA) and catalase (CAT), glutathione peroxidase (GPx) (Cayman Chemical, MI, USA), superoxide dismutase (SOD) (Arbor Assays, MI, USA), and glutathione reductase (GR) (Biovision, CA, USA) activities were measured in plasma by colorimetric assays following the manufacturer’s instructions. Absorbance values were measured with the CLARIOstar plus microplate reader (BMG Labtech, Ortenberg, Germany). Plasmatic levels of oxLDL were measured by the mouse oxidized low density lipoprotein (oxLDL) ELISA kit (MyBioSource, CA, USA) according to the manufacturer’s indications. Absorbance values at 450 nm were measured with the Multiskan FC microplate photometer (Thermo Scientific). The representative standard curves are showed in Supporting Information, Figure S1, and the sensitivity and inter/intra assay precision are shown in Supporting Information, Table S2.

### cDNA Synthesis and RT qPCR

RNA was obtained from the aorta artery using TRIsure (Bioline). Reverse transcription of 3 μg of RNA was carried out using the Transcriptor First Strand cDNA Synthesis Kit (Roche). qPCR (80 ng cDNA/well) was performed by the SYBR Green I Master kit and the LightCycler 480 thermocycler (all from Roche). The reference gene hypoxanthine phosphoribosyl transferase (*hprt*) was used to calculate the relative gene expression using the 2^–ΔΔCt^ method. In Table S3 of Supporting Information are reported primers sequences and RT-PCR conditions.

### Cell Culture

The fresh aorta artery was digested with collagenase type XI (125 U/mL) (Sigma-Aldrich), collagenase type I (450 U/mL) (Worthington, NJ, USA), hyaluronidase type 1-s (60 U/mL) (Sigma-Aldrich), and DNase I (60 U/mL) (PanReac AppliChem, IL, USA) in PBS with HEPES 20 mM for 1 h at 37 °C in agitation. The digested tissue was then passed through a 70 μm mesh and the resulting was cultured at 4 × 10^6^ cells/mL in RPMI 1640 medium supplemented with 5% fetal bovine serum, 1% glutamine, and 1% penicillin/streptomycin (all from BioWest, Nuaillé, France). Cells were cultured at 37 °C in a humidified atmosphere with 5% CO_2_.

### Flow Cytometry

After cultured overnight, aortic cells were incubated with brefeldin A (3 μg/mL, eBioscience, San Diego, CA, USA) for the last 5 h of culture and with or without phorbol 12-myristate 13-acetate (0.5 μg/mL)/ionomycin (1 μg/mL). Subsequently, the cells were stained with extracellular anti-CD4 and anti-CD11b antibodies. After fixation/permeabilization with the BD Cytofix/Cytoperm Fixation/Permeabilization Solution Kit (BD Biosciences), an intracellular labeling was performed for interferon-γ (IFN-γ) cytokine. Viability was measured using the LIVE/DEAD Fixable Dead Cell Stain Kit (Invitrogen, MA, USA) to exclude dead cells from the analyses. Flow cytometry measurements were performed using the BD LSRFortessa cell analyzer (BD Bioscience) and data were analyzed using FlowJo software (TreeStar, OR, USA). The characteristics of flow cytometry antibodies are described in Supporting Information, Table S4.

### Cytokines and Chemokines Quantification

Cytokines [interleukin (IL)-1β, IL-4, IL-5, IL-6, IL-17, IL-18, IL-23, IFN-γ, tumor necrosis factor (TNF), and granulocyte-macrophage colony-stimulating factor (GM-CSF)] and chemokines [Eotaxin, RANTES, chemokine (C-X-C motif) ligand 1 (CXCL1), interferon γ-induced protein (IP)-10, monocyte chemoattractant protein (MCP)-1, MCP-3, macrophage inflammatory protein (MIP)-1α, MIP-1β, and MIP-2] were quantified in 48 h supernatant of aortic cell cultured stimulated with 8 μg/mL phytohemagglutinin-P (PHA) (Sigma-Aldrich) using the ProcartaPlex Multiplex Immunoassay (Invitrogen), a bead-based multiplex immunoassay, following the manufacturer’s instructions. The signal was recorded by the Luminex Bio-Plex 200 System (Bio-Rad, CA, USA) and data were analyzed with ProcartaPlex Analyst Software (Invitrogen).

### Proliferation Assay

Cell proliferation was determined in PHA-stimulated cells cultured for 36 h using the BrdU Cell Proliferation ELISA Kit (Roche), a colorimetric immunoassay. A Multiskan FC microplate photometer (Thermo Scientific) was employed to measure the absorbance (450 nm).

### Statistical Analysis

Data were expressed as mean ± standard error of the mean. Differences between groups were evaluated by the unpaired and nonparametric Mann–Whitney *U* test using the SPSS software v24 (IBM Corporation, NY, USA). Values of *p* ≤ 0.05 were considered statistically significant.

## Results

### LPH Characterization

**Figure 1:**
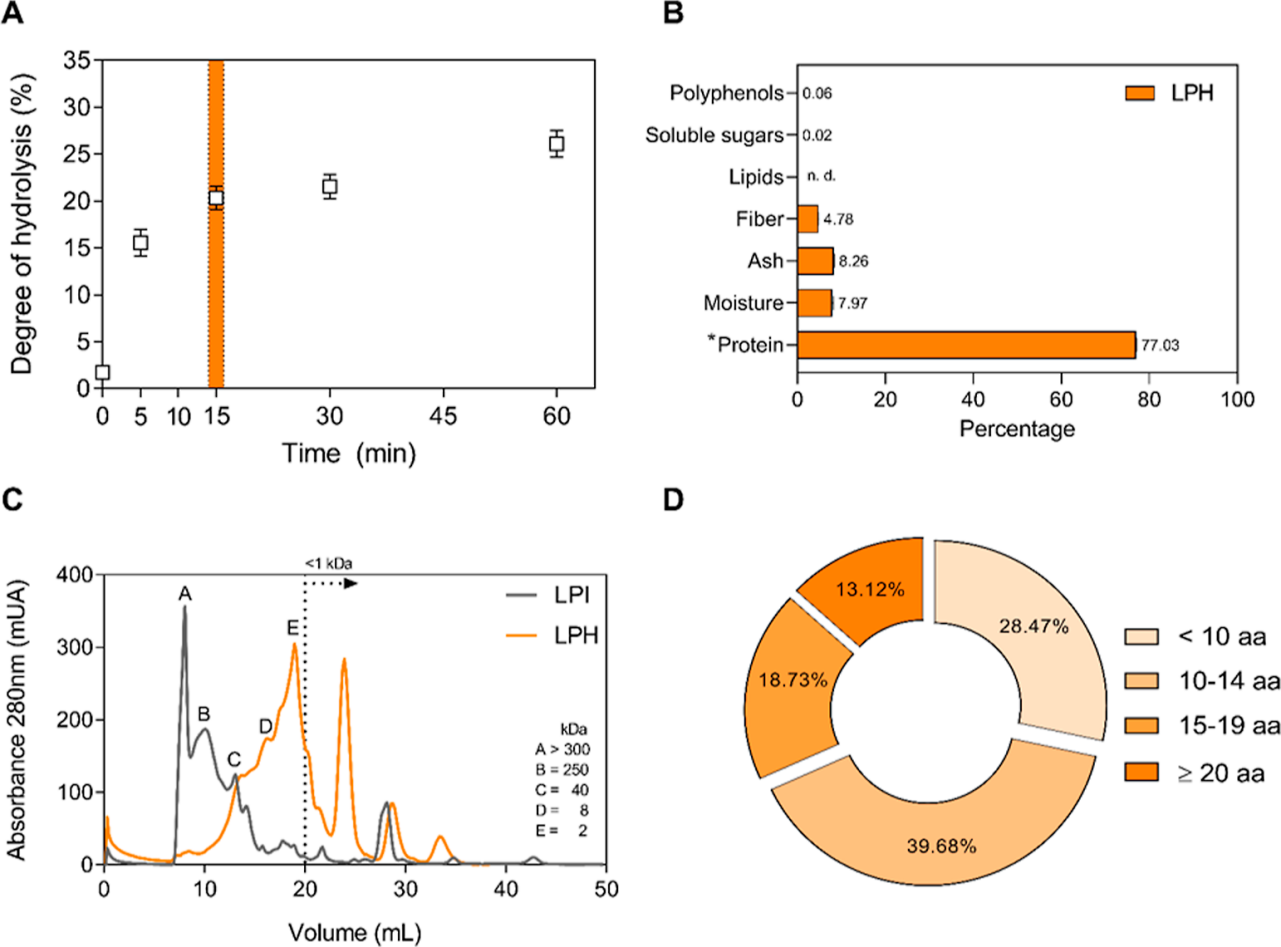
Degree of hydrolysis vs time obtained during Alcalase hydrolysis of LPI (A), chemical composition of LPH (B), molecular weight profile of LPI and LPH (C), and length distribution of the peptides from LPH (D). Data are expressed as mean ± standard deviation. aa, amino acids; LPH, lupin protein hydrolysate; LPI, lupin protein isolate; n.d.: no detected; *, calculated as nitrogen (N) × 6.25.

The Alcalase enzyme was used at different time-points to obtain the best degree of hydrolysis of LPI. As shown in Figure 1A, the degree of hydrolysis was 15.56 ± 1.40% at 5 min, 20.30 ± 1.27% at 15 min, 21.54 ± 1.30% at 30 min, and 26.13 ± 1.40% at 60 min. Hydrolysis for 15 min was selected to obtain a high percentage of hydrolysis in the shortest time. To characterize the LPH, the molecular weight profile and the chemical and amino acid composition were studied in the dry matter. LPH was mainly composed of proteins (77.03 ± 0.09%, Figure 1B), with higher levels of Glu/Gln, Arg, and Asp/Asn, and lower levels of Pro, Cys, Met, and Trp (amino acid composition is shown in Supporting Information, Table S5). Figure 1C shows the molecular weight profile of LPI and LPH. Protein size of LPI was between 40 to >300 kDa, with the highest peak at >300 kDa. On the contrary, LPH peptide size was between 40 to <1 kDa, with the highest peak in <2 kDa. Furthermore, the set of peptides that constituted the LPH consisted of 2–26 amino acid residues, being the most frequent peptides (68.15%) those containing between 2 and 14 amino acid (Figure 1D).

### LPH Sequences Identification with Biological Activities

278 peptides from LPH were identified as specific lupine-derived peptides by the Proteome Discoverer software using the database downloaded from UniProt (https://www.uniprot.org) for the taxonomy*L. angustifolius*.^22^ These peptides possessed molecular weights between 755.5 and 3111.5 Da, and the length of these ranged from 7 to 26 amino acids. As it is shown in Table 1, of the 278 peptides identified, 259 (93.16%) presented motifs with already demonstrated lipid-lowering (2 sequences, 0.72%), antioxidant (165 sequences, 59.35%), immunomodulatory (22 sequences, 7.91%), and antithrombotic activities (70 sequences, 25.08%) according to the BIOPEP-UWM database.^30^

**Table 1:** Number of LPH Sequences with Biological Activities Related to Atherosclerosis Risk Factors

biological activity	ID^a^	bioactive motif^b^	no. sequences	total sequences
hypolipidemic	9580	EF	1	2
	9384	IVG	1	
antioxidative	3300	PHH	1	165
	3301	HLH	1	
	3305	LH	5	
	3314	LLPH	9	
	3317	HL	7	
	3319	HH	7	
	7862	IKK	1	
	7866	AY	4	
	7872	LY	2	
	7886	AH	4	
	7888	EL	12	
	7898	WY	1	
	7918	GHH	1	
	7980	HHH	1	
	7995	LHL	4	
	8001	LHT	1	
	8038	PHY	8	
	8045	PWL	1	
	8053	PWY	1	
	8063	RHN	2	
	8065	RHR	3	
	8076	RWL	1	
	8103	VKL	1	
	8107	IKL	3	
	8114	GGE	1	
	8133	KVI	5	
	8134	KD	4	
	8190	PW	2	
	8214	RW	1	
	8215	IR	28	
	8216	LKP	1	
	8217	LK	15	
	8218	KP	2	
	8219	TY	1	
	8224	VY	1	
	8459	TW	1	
	8461	VW	1	
	9879	SVL	1	
	9537	IPP	3	
	9538	VPP	6	
	10000	LPL	5	
	10003	LQL	1	
	10051	RY	5	
immunomodulating	2882	YG	2	22
	3626	KRP	1	
	9856	PY	8	
	9869	HY	10	
	9870	LPF	1	
antithrombotic	283	GP	8	70
	3284	PGP	5	
	3285	PG	48	
	3354	DEE	9	
				259

a According to the BIOPEP-UWM database.^30^

b 1-Letter amino acid code.

### LPH Treatment Does Not Change Calorie Intake and Body Weight

To evaluate the effect of LPH consumption on body weight, a daily control of mice weight was carried out in both experimental groups (Ctrl and LPH). As shown in Table 2, the initial body weight (IBW), as well as the final body weight (FBW) were not modified by LPH-treatment. Additionally, the body weight gain (BWG) and the daily food intake (DFI) were not altered between the experimental groups. The body weight evolution throughout the experiment is represented in Supporting Information, Figure S2.

**Table 2:** Body Weight Parameters and Daily Food Intake^a^

parameter	Ctrl	LPH
IBW (g)	21.18 ± 0.30	21.23 ± 0.27
FBW (g)	28.77 ± 0.68	28.73 ± 0.65
BWG (g)	7.59 ± 0.48	7.28 ± 0.45
DFI (g/mouse)	2.46 ± 0.03	2.44 ± 0.05

a Initial body weight (IBW), final body weight (FBW), body weight gain (BWG), and daily food intake (DFI) in ApoE^–/–^ mice. Values are shown as the mean ± standard error of the mean of each group (*n* = 30 per group). Ctrl, control group. LPH, lupin protein hydrolysate group.

### LPH Administration Decreases the Plasmatic Lipid Concentration

Plasmatic TC, LDL-C, and TG concentrations of LPH-treated mice were significantly reduced with respect to the control group, without alteration in the HDL-C concentration (Table 3). Furthermore, CRI (I), CRI (II), and AIP decreased significantly with the LPH treatment (Table 3).

**Table 3:** Plasma Lipid Profile^a^

parameters	control	LPH	difference (%)	*p*-value
TC (mg/dL)	420 ± 14	370 ± 16	–11.90	**0.027**
TG (mg/dL)	133 ± 6	113 ± 4	–15.04	**0.024**
HDL-C (mg/dL)	48 ± 2	50 ± 2	+4.17	0.984
LDL-C (mg/dL)	328 ± 14	295 ± 14	–10.06	**0.026**
CRI (I)	8.35 ± 1.30	7.50 ± 1.14	–10.18	**0.012**
CRI (II)	8.51 ± 2.62	7.67 ± 2.51	–9.87	**0.025**
AIP	0.412 ± 0.09	0.344 ± 0.07	–16.50	**0.017**

a Mean ± SD of the absolute lipid profile values of the two experimental groups (*n* = 20 per group), and the difference between them expressed in percentage. AIP, atherogenic index of plasma [Log_10_(TG/HDL-C)]; CRI, Castelli risk index (CRI I: TC/HDL-C; CRI II: LDL-C/HDL-C); HDL-C, high-density lipoprotein cholesterol; LDL-C, low-density lipoprotein cholesterol; LPH, lupin protein hydrolysate group; TC, total cholesterol; TG, triglycerides.

### LPH Treatment Improves the Plasma Antioxidant Status

**Figure 2:**
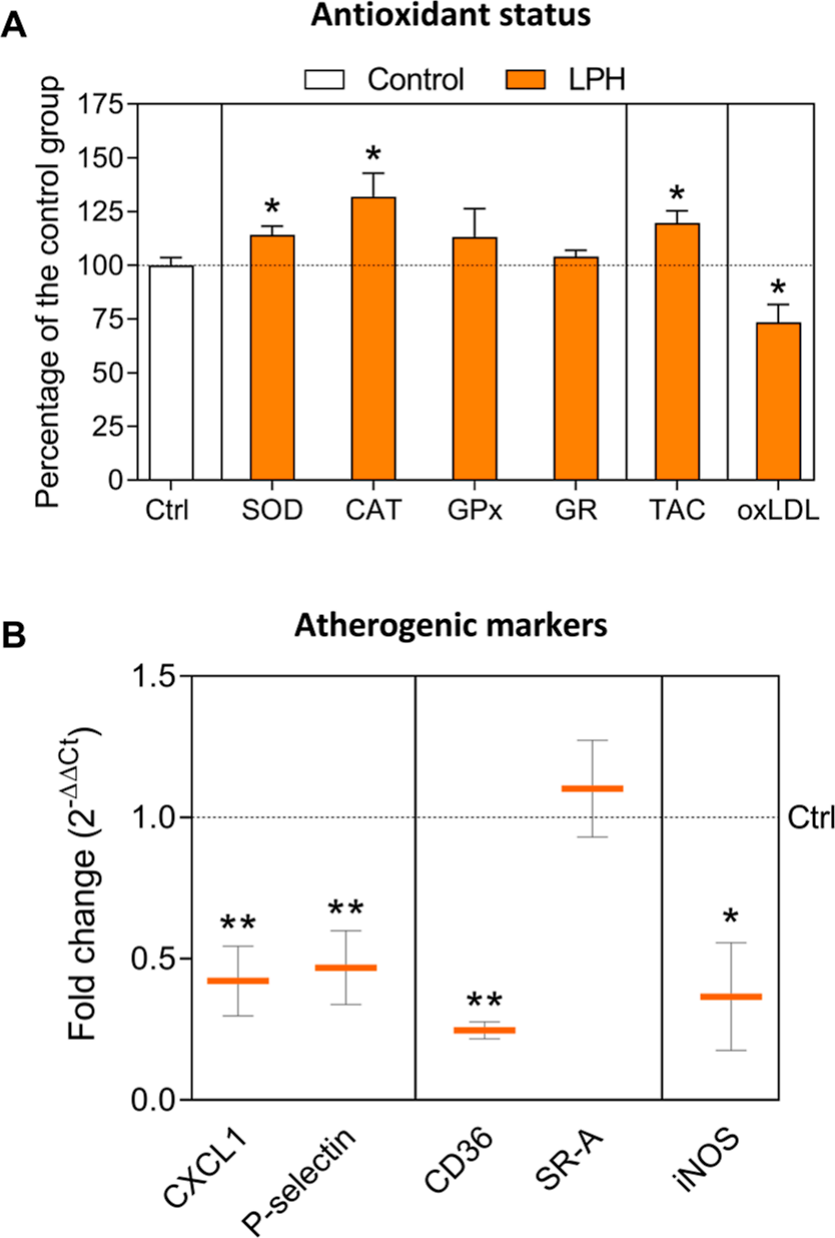
Antioxidant status (A): CAT, SOD, GPx, and GR antioxidant activities, TAC, and oxLDL levels in the plasma of ApoE^–/–^ mice after 12 weeks of treatment with LPH. Results are expressed as a percentage of the control group and represent the mean and standard error of the mean of each group (*n* = 15 per group). Atherogenic markers gene expression (B): relative gene expression of chemoattractant (CXCL1), adhesion molecule (P-selectin), scavenger receptors (CD36, and SR-A), and iNOS in the aorta of mice after 12 weeks of treatment with LPH. Data are shown as the mean of 2^–ΔΔCt^ and standard error of the mean of each group (*n* = 7 per group). *p ≤ 0.05, **p ≤ 0.01 with respect to the control group. CAT, catalase; CD36, cluster of differentiation 36; CXCL1, C-X-C motif chemokine ligand 1; GPx, glutathione peroxidase; GR, glutathione reductase; iNOS, inducible nitric oxide synthase; LPH, lupin protein hydrolysate group; oxLDL, oxidized low-density lipoprotein; SelP, P-selectin; SOD, superoxide dismutase; SR-A, class A macrophage scavenger receptor.

An LPH-mediated significant increase of the enzymatic activities of SOD and CAT was observed in the plasma (Figure 2A). Neither GPx nor GR activities were significantly affected by the LPH treatment (Figure 2A). Furthermore, a significant increase in plasma TAC of LPH-treated mice was detected (Figure 2A). According to the improvement in LPH-induced plasma antioxidant status, oxLDL levels were significantly reduced after LPH treatment (Figure 2A). Raw data are reported in Supporting Information, Table S6.

### LPH Reduces the Gene Expression of Atherogenic Markers

To determine the anti-atherogenic effects of LPH, the mRNA levels of key atherogenic markers were quantified in the aorta artery. LPH treatment significantly reduced the relative expression of CXCL1, P-selectin, cluster of differentiation (CD) 36, and inducible nitric oxide synthase (iNOS) mRNA (Figure 2B).

### LPH Treatment Reduces Aortic Inflammation and Promotes an Anti-Atherogenic Microenvironment

**Figure 3:**
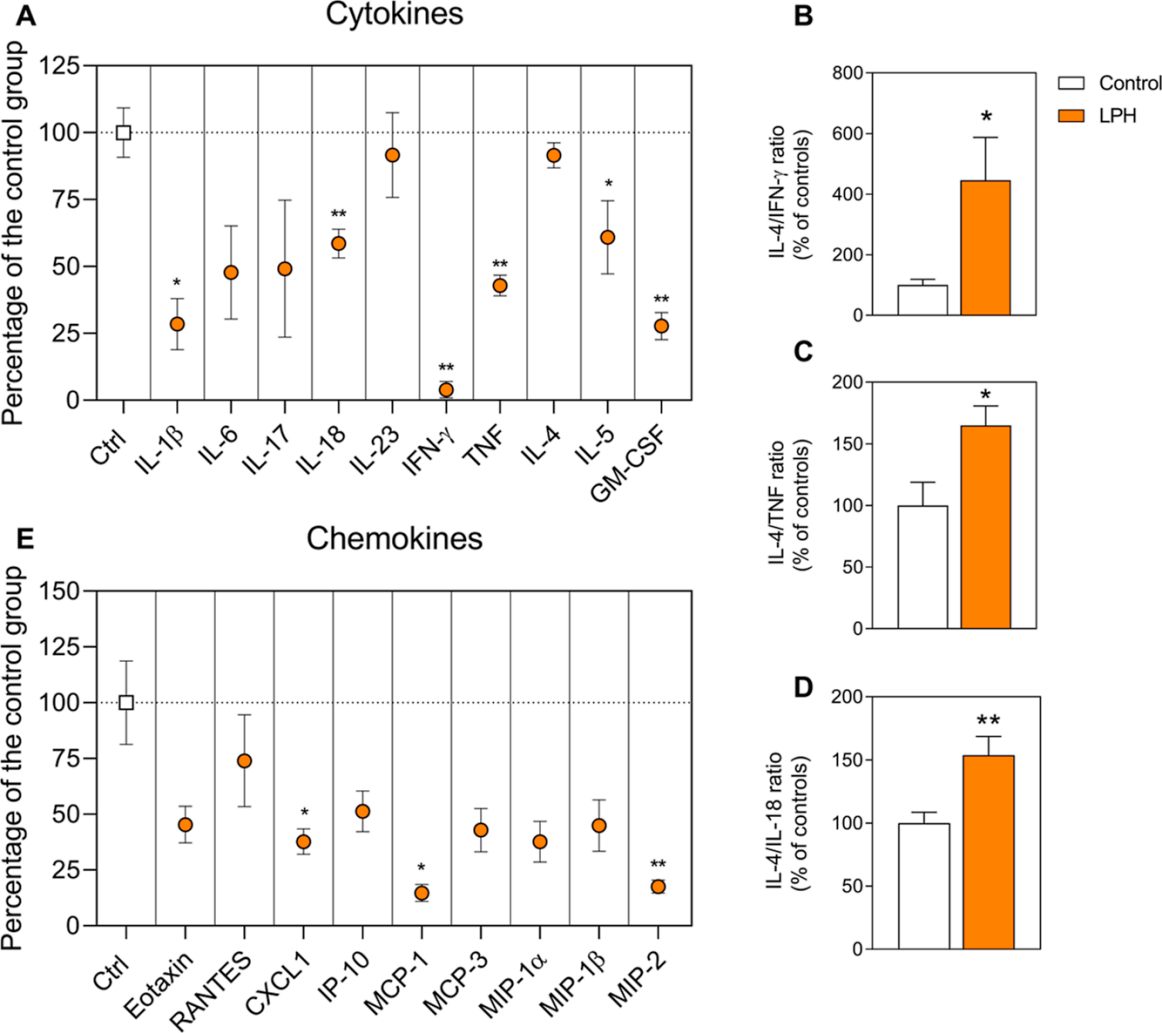
Pro- and anti-inflammatory cytokines (A) and chemokines (E) production in PHA-stimulated aortic cells. Ratios between IL-4 and pro-inflammatory cytokines IFN-γ (B), TNF (C), IL-18 (D). The results are expressed as the percentage of the control group and represent the mean and standard error of the mean (*n* = 12 per group). *p ≤ 0.05; **p ≤ 0.01. CXCL1, C-X-C motif chemokine ligand 1; GM-CSF, granulocyte-macrophage colony-stimulating factor; IFN-γ, interferon-γ; IL: interleukin; IP-10, IFN-γ inducible protein-10; LPH, lupin protein hydrolysate group; MCP, monocyte chemoattractant protein; MIP, macrophage inflammatory protein; PHA, phytohemagglutinin-P; RANTES, regulated on activation normal T cell expressed and secreted; and TNF, tumor necrosis factor.

To analyze the inflammatory status in the aorta cells, cytokines and chemokines were quantified in the cell culture supernatants. IL-1β, IL-18, IFN-γ, and TNF [pro-inflammatory T helper (Th) 1 cytokines] were significantly decreased by the LPH treatment in comparison to the control group (Figure 3A). On the contrary, LPH treatment did not affect the Th2 anti-inflammatory cytokine (IL-4) production (Figure 3A). Interestingly, ratios of IL-4/IFN-γ, IL-4/IL-18, and IL4/TNF were significantly increased in the LPH group (Figure 3B–D).

Furthermore, pivotal chemokines implicated in atherogenesis (CXCL1, MCP-1, and MIP-2) (Figure 3E) and GM-CSF (Figure 3A) were decreased in LPH-treated mice. Additionally, the production of the IL-6 inflammatory cytokine (Figure 3A) and pro-inflammatory chemokines (MIP-1α, MIP-1β, MCP-3, and IP-10) (Figure 3E) showed a trend to decrease in the aortic cells from LPH-treated mice.

To verify that the decrease in the cytokines and chemokines concentration was not due to the cytotoxic or anti-proliferative effects of LPH, cellular viability and proliferation were assayed. Neither toxic nor anti-proliferative effects were observed in LPH with respect to the untreated group (Supporting Information, Figure S3).

**Figure 4:**
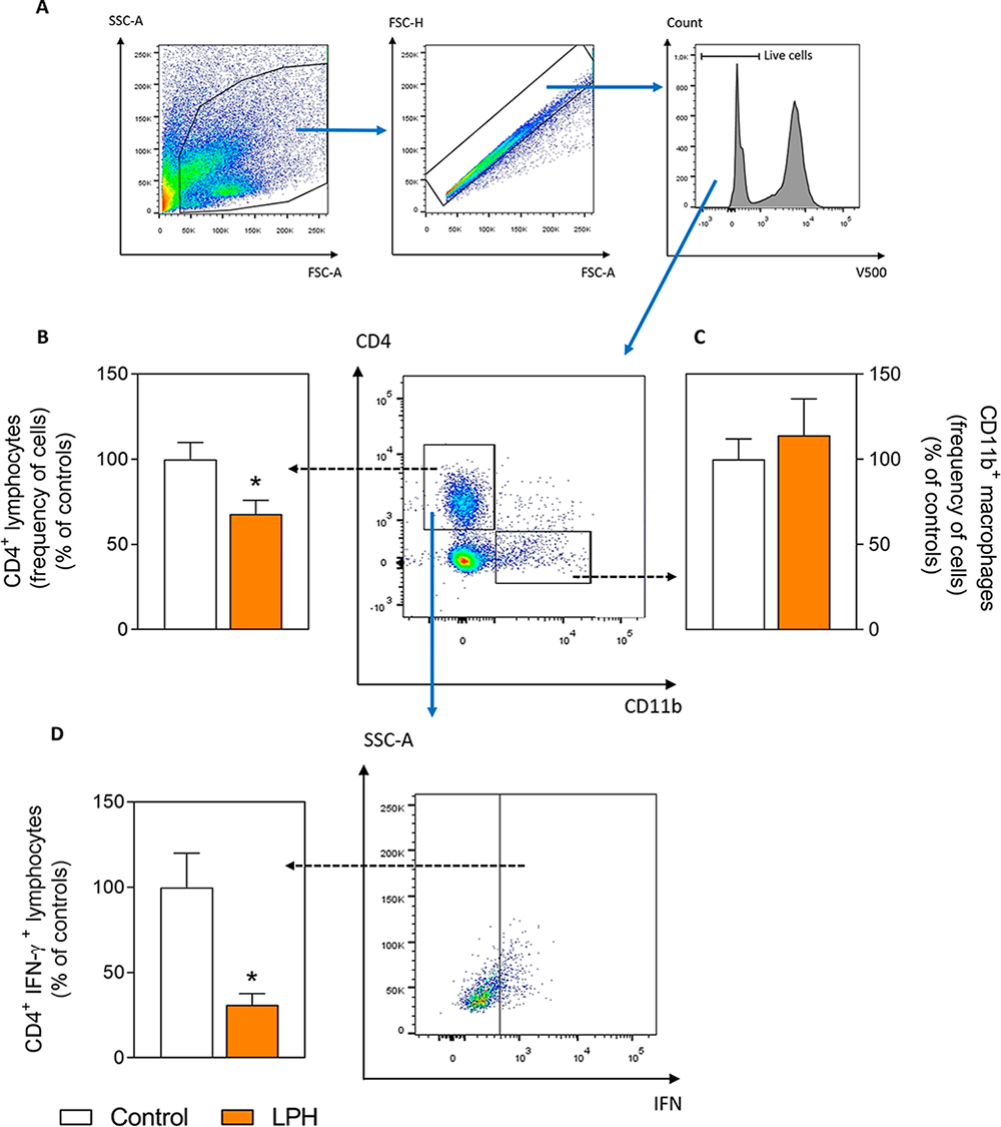
Schematic representation of gate-strategy of aorta artery cells in the flow cytometry experiments (A). Lymphocytes (CD4^+^ cells) (B), macrophages (CD11b^+^ cells) (C), and CD4^+^ cells producing the IFN-γ pro-inflammatory cytokine (CD4^+^ IFN-γ^+^ cells) (D) of PHA-stimulated aortic cells (*n* = 10 per group). Results are expressed as a percentage of the control group and represent the mean and standard error of the mean of each group. *p ≤ 0.05, **p ≤ 0.01 with respect to the control group. IFN-γ, interferon-γ; IL, interleukin; LPH, lupin protein hydrolysate group; PHA, phytohemagglutinin-P; and TNF, tumor necrosis factor.

The LPH effect on the aortic inflammatory infiltration was explored by the T lymphocytes (CD4^+^ cells) and macrophages (CD11b^+^ cells) quantification (schematic representation of gate selection is shown in Figure 4A). The CD4^+^ T lymphocytes frequency was significantly reduced by the LPH treatment (Figure 4B), while no differences were observed in CD11b^+^ macrophages (Figure 4C). LPH treatment not only reduced the percentage of CD4^+^ T lymphocytes in the aorta, but also significantly decreased the frequency of pro-inflammatory CD4^+^ T cells producing IFN-γ (Figure 4D).

## Discussion

In recent years, bioactive peptides have attracted considerable attention as effective dietary sources.^13^ Enzymatic hydrolysis is the major tool to generate bioactive peptides in a sustainable way maintaining the nutritional and functional quality of the products generated compared to chemical hydrolysis.^34^ Additionally, the hydrolysis process can cleave those epitopes involved in the allergic process of lupin, generating hypoallergenic products that could be suitable for people allergic to lupin.^35,36^ In this work, we have used the enzyme Alcalase enzyme to generate the LPH. Alcalase is an endopeptidase that cleave peptides bond from a wide range of amino acids (Glu, Met, Leu, Tyr, Lys, and Gln) giving rise to protein hydrolysates with high peptide content of small size with an optimal hydrolytic activity within pH 7–10 and 50–70 °C.^37^

LPH characterization showed that the protein content (nitrogen content × 6.25) was 77.03 ± 0.09%, much higher than the starting flour (30–40%). Furthermore, the amino acid content of LPH was balanced, according to the WHO and FAO recommendations, containing significantly low values in sulfur amino acids, an innate characteristic of legumes species. In addition, the use of the Alcalase enzyme allowed us to generate low-molecular-weight peptides in comparison with the LPI protein. This aspect is interesting because it is well known that the biological activity is higher when the peptides are small.^34^ Specifically, LPH showed to contain peptides below 2 kDa. The in silico analysis of the LPH peptides revealed the presence of 278 sequences with previously described bioactivity. The analysis of these set of peptides allowed us to identify 259 sequences (93.16%) containing motives with biological activities related to atherosclerosis risk factors (hypolipidemic, antioxidant, immunomodulating, and antithrombotic).

The *in vivo* evaluation showed the beneficial effects of 12 weeks’ treatment with LPH in a preclinical model of WD-induced atherosclerosis in ApoE^–/–^ male mice. LPH reduced hyperlipidemia, oxidative stress, and inflammation, which are key components of the early stage of atherosclerosis and the further disease processes.^5,6,38^ LPH was shown to have two action mechanisms. At the systemic level, LPH reduced blood lipids and increased plasma antioxidant capacity. Furthermore, LPH directly influenced aorta by decreasing endothelial permeability, the inflammatory response of immune-infiltrating cells, and the level of key atherogenic markers.

To study the early events of atherosclerosis, we used a short-term design (12 weeks) and a diet with 45% fat energy and a normal cholesterol concentration. On the contrary, a diet richer in fat energy (usually 60%) and cholesterol, as well as long-term treatment, is more appropriate to study the final stages of atheroma plaque generation (commonly called late atherosclerosis).^39^ This is possible due to ApoE^–/–^ mice have been shown to develop the entire spectrum of all steps of atherosclerosis, including the early stages depending on the type of diet and the duration of the consumption.^7,31,32,40^

LPH-treated mice showed not only lower plasma concentrations of TC, LDL-C, and TG with respect to the untreated control group but also a significant reduction of CRI (I), CRI (II), and AIP. This is of special interest given that these indexes are used as optimal indicators of cardiovascular risk, and are considered more sensitive and specific than individual lipid parameters, both in humans and mice.^10,41,42^ Interestingly, the effect of LPH on blood lipid lowering was not related to diet intake or BWG that were not altered between groups. Although other authors have described that whole lupin protein exert a lipid-lowering effect in animals and humans,^43,44^ we are not aware of previous studies demonstrating the wide range of hypolipidemic effects of lupin protein hydrolysates related to atherosclerosis described in the present work.

Furthermore, LPH increased plasma SOD and CAT enzymatic activities, as well as TAC, which is in agreement with our previous studies on human cells.^20^ Additionally, the ingestion of a functional beverage based on LPH during 4 weeks also increases the TAC in human cells from healthy volunteers in the lupine-1 trial, conducted by our group.^23^ Guo et al. have also shown that LPH, produced by Alcalase digestion (pH 10, 50 °C, 240 min), increases the enzymatic activities of SOD and GPx in the human hepatocyte cell line, HepG2.^45^ Given that ApoE^–/–^ mice overexpressing both the SOD and CAT enzymes have a significant reduction in atherogenic lesions,^46^ our results showing the activating role of LPH in both antioxidant enzymes are of special interest. Furthermore, LPH treatment significantly reduced circulating oxLDL levels. The supplementation of diet with 25 g/d of lupin proteins for 28 d in hypercholesterolemic subjects has shown no effect on blood oxLDL concentration,^44^ suggesting that the bioactivity of LPH observed could come from the hydrolysis process that generates small peptides, which are in fact more bioactive than the whole protein.^13^ The effects of LPH on LDL-C and oxLDL are remarkable because the atherosclerosis has been shown to be initiated as an inflammatory response from the arterial subendothelial compartment due to the accumulation of LDL-C and oxLDL that involves an increase in endothelial permeability driven by the overproduction of cell adhesion molecules, chemoattractants, and inflammatory molecules.^1^ In this way, we showed a significant decrease in P-selectin gene expression in the aorta of LPH-treated mice. This is of special interest, given that this selectin promotes atherosclerosis by increasing leukocyte infiltration at sites of endothelial injury.^47^ Indeed, a positive correlation has been reported between P-selectin gene expression and the development of atherosclerotic lesions.^48^ Furthermore, some authors have shown that the knockout of P-selectin reduces the atheroma plaque area in ApoE^–/–^ mice fed WD,^49^ considering P-selectin as a therapeutic target in atherosclerosis.^48^ Chemokines (MCP-1, CXCL1, and MIP-2), which are implicated in atherosclerosis by inducing leukocyte trafficking to the inflamed site, were reduced in the aortic cells by the LPH treatment.^50^ In this line, MCP-1 absence reduces aortic leukocyte infiltration in the ApoE^–/–^ mouse model.^51^ MCP-1 also promotes the ligand–receptor interaction of oxLDL-CD36,^52^ an essential process in the foam cells formation because CD36 is the principal surface receptor for oxLDL. Interestingly, we showed a considerable reduction in CD36 gene expression levels in the aorta of LPH-treated mice. Therefore, the combined actions in MCP-1, oxLDL, and CD36 suggest a beneficial role for LPH in foam cell development. Resident macrophages in the vascular intima of the aorta also have the ability to indiscriminately phagocytize oxLDL by the surface scavenger receptor (SR)-A.^9^ The aorta of animals treated with LPH do not show changes in the expression of the SR-A gene. In this regard, some studies have reported that the absence of CD36, but not SR-A, results in a reduction in the area of the aorta lesion in ApoE^–/–^ mice.^53^ Treatment with LPH also decreased the expression of the aortic CXCL1 chemokine, which allows the neutrophils recruitment to the site of inflammation.^50,54^ Both CXCL1 and MIP-2 have been associated with the development of atherosclerosis and cardiovascular diseases.^54^ In addition to controlling chemoattractant agents, LPH treatment also decreased GM-CSF production, a myeloid growth factor involved in monocyte differentiation to inflammatory macrophages.^55^ The pro-atherogenic role of GM-CSF has also been shown in ApoE^–/–^ mice.^56^

Considering the above findings, and that this work is the first to show the beneficial effects of LPH on endothelial dysfunction, we evaluated the possible function of LPH in the modulation of leukocytes recruitment to the aorta. LPH treatment decreased not only the frequency of infiltrating CD4^+^ cells but also their capacity to produce the Th1 pro-inflammatory cytokine IFN-γ. According to this anti-inflammatory effect, the LPH treatment improved the ratio of IL-4 (anti-inflammatory Th2 cytokine) and Th1 cytokines (IFN-γ, TNF, and IL-18) in the aortic cells. Interestingly, no effect of LPH was observed on the frequency of aortic macrophages. However, treatment with LPH reduced the aortic concentration of pro-inflammatory cytokines (IL-1β, IL-18, and GM-CSF) and chemokines (CXCL1 and MIP-2) produced primarily by macrophages, suggesting the capacity of LPH to modulate the effector function of macrophages. In this way, IFN-γ and TNF can also be produced by macrophages. Thus, the combined action of LPH in the effector capacity of both CD4^+^ cells and macrophages confirms the anti-inflammatory micro-environment in the aorta of LPH-treated mice. These facts are noteworthy given that several authors have shown the relevance of pro-inflammatory mediators in atherosclerotic lesion formation progression.^2,3^ Although lupin-derived peptides exert *in vitro* activity on Th1 cytokine production,^20^ this is the first description of an *in vivo* anti-inflammatory role of LPH related to arterial dysfunction and atherosclerosis. LPH also diminished the aortic iNOS gene expression. iNOS produces nitric oxide (NO), which is an important compound implicated in the inflammation and in the formation of atherosclerotic lesions.^57^ To our knowledge, no previous reports have shown the *in vivo* action of lupin-derived peptides on the expression of iNOS.

**Figure 5:**
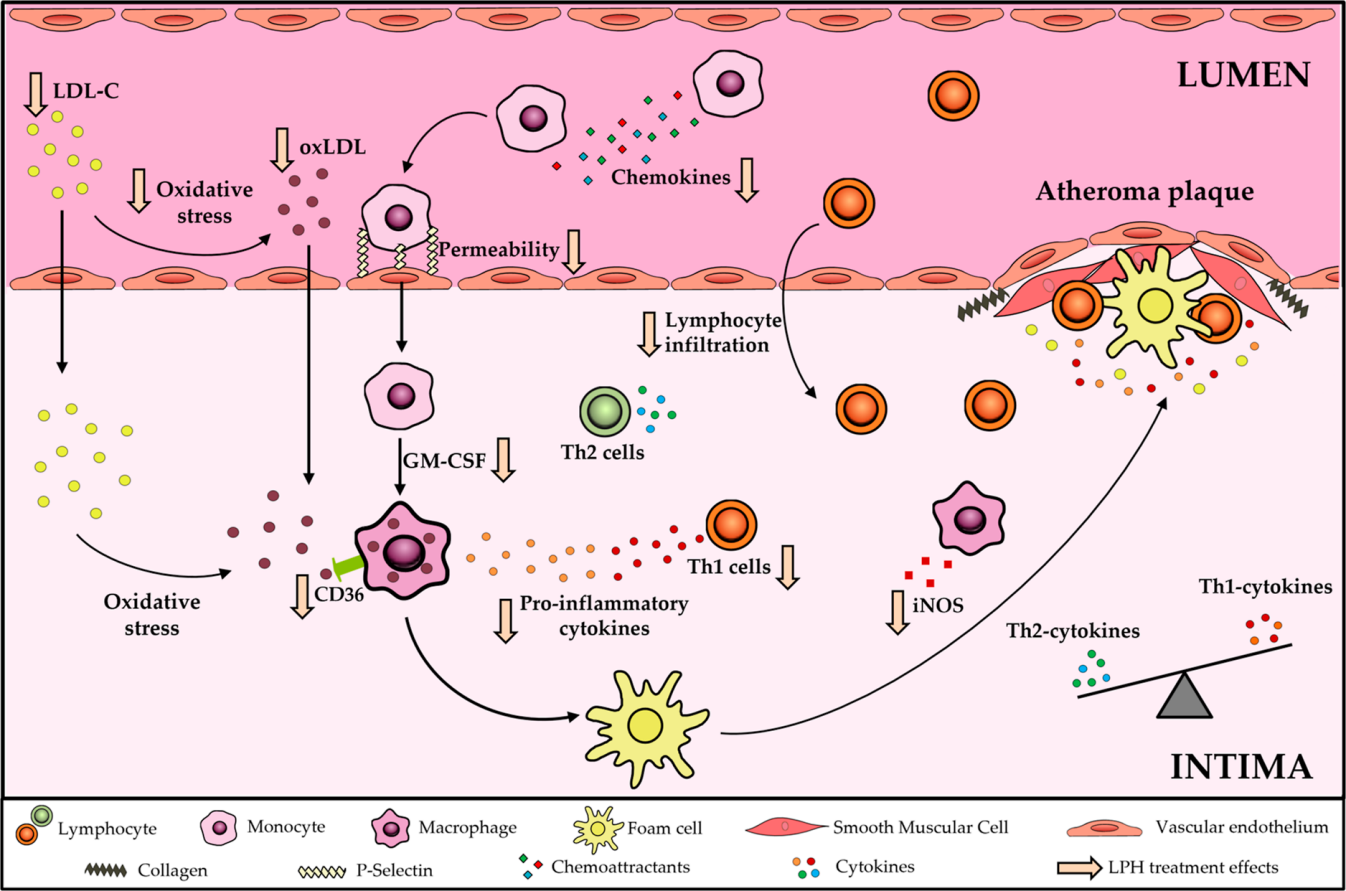
Summary of the LPH atheroprotective effects; arrows show the effects of the LPH observed in this study. CD36, cluster of differentiation 36; LDL-C, low-density lipoprotein cholesterol; SR-A, class A macrophage scavenger receptor, LPH, lupin protein hydrolysate; and oxLDL, oxidized low-density lipoprotein.

In conclusion, although previous studies have shown that LPI can decrease the calcification of atherosclerotic lesions in ApoE knockout mice,^14,58^ the present study shows the multifunctional properties of the peptides from lupin in the key steps of atherosclerosis. LPH, produced by hydrolysis with Alcalase, contained peptides with several motifs associated with hypolipidemic, antioxidant, immunomodulating, and antithrombotic effects. Moreover, LPH treatment reduced the plasma lipid content, oxidative stress, and aortic inflammation (summarized in Figure 5), all key risk factors for atherosclerosis development. Therefore, this study points out LPH as a promising ingredient for the improvement of nutraceuticals or functional foods.

## Supporting Information Available

Composition of experimental WD; sensitivity and inter/intra assay precision of the assays used in this study; primer sequences and qPCR conditions; flow cytometry antibodies characteristics; amino acid composition of LPH; raw data of antioxidant parameters; representative standard curve of the antioxidant assays used in this study; body weight monitored over time; and cell proliferation and cell viability (PDF)

## Abbreviations

AIP: atherogenic index of plasma
CAT: catalase
CD: cluster of differentiation
CRI: Castelli risk index
CXCL1: chemokine (C-X-C motif) ligand 1
GM-CSF: granulocyte-macrophage colony-stimulating factor
GPx: glutathione peroxidase
GR: glutathione reductase
IP-10: IFN-γ inducible protein-10
LPH: lupin protein hydrolysate
MCP: monocyte chemoattractant protein
MIP: macrophage inflammatory protein
SOD: superoxide dismutase
SR-A: scavenger receptor A
TAC: total antioxidant capacity
