# Supplementary material for: Bioactive Peptides from Lupin (Lupinus angustifolius) Prevent the Early Stages of Atherosclerosis in Western Diet-Fed ApoE–/– Mice
Source: J Agric Food Chem. 2022 Jun 29;70(27):8243–53. doi: 10.1021/acs.jafc.2c00809 (PMC9284549; doi:10.1021/acs.jafc.2c00809)
Supplement: Supplementary file 1 — jf2c00809_si_001.pdf [file jf2c00809_si_001.pdf]

Bioactive peptides from Lupin (*Lupinus angustifolius*) prevent the early stages of atherosclerosis in Western diet-fed ApoE$^{-/-}$ mice

Guillermo Santos-Sánchez, Ivan Cruz-Chamorro*, Ana Isabel Álvarez-Ríos, Nuria Álvarez-Sánchez, Beatriz Rodríguez-Ortiz, Ana Isabel Álvarez-López, María-Soledad Fernández-Pachón, Justo Pedroche, Francisco Millán, María del Carmen Millán-Linares, Patricia Judith Lardone, Ignacio Bejarano, Antonio Carrillo-Vico*

***** Corresponding authors: Instituto de Biomedicina de Sevilla, IBiS (Universidad de Sevilla, HUVR, Junta de Andalucía, CSIC), 41013 Seville, Tel: +34955923106. vico@us.es (A.C.-V.); icruz-ibis@us.es (I.C.-C.).

Lupin protein hydrolysate preparation

Lupin protein hydrolysate (LPH) was obtained at the Instituto de la Grasa (CSIC, Seville, Spain). Lupin protein isolate (LPI) suspended in distilled water (10% w/v) and was hydrolysed in a bioreactor using the followed conditions: 15 minutes with Alcalase 2.4L enzyme (2.4 AU/g; Novozymes, Bagsvaerd, Denmark) at pH = 8 (maintained throughout the process by adding 1N NaOH), temperature of 50 °C, and E/S = 0.3 AU/g protein. To inactivate the enzyme, the temperature was increased up to 85 °C for 15 minutes and the subsequent centrifugation (10,437 g for 15 minutes) allowed for to collection of the supernatant (= LPH). The latter was lyophilized and stored at room temperature.[1]

Proteins concentration

Concentration of proteins was assessed by the Leco CN 828 analyzer (St. Joseph, MI, USA) as a percentage of nitrogen content × 6.25. Heating for 25 h at 550 °C allowed for to calculation of the ash content.

Degree of hydrolysis

The degree of hydrolysis was calculated according to the TNBS method.[2] To quantify the total number of amino groups, a 100% hydrolysed specimen was used (110 °C for 24 h in 6 N HCl).

Molecular weights profile

Molecular weights profile of the LPH was carried out by molecular exclusion gel filtration chromatography in an Akta Purifier system (GE Healthcare) equipped with a Superose 12 HR 10/30 column with an optimal separation range of 300 – 1,000 Da. The elution was developed in 50 mM sodium phosphate 0.5 M NaCl, 0.02% (w/v) sodium azide

buffer and monitored at 280 nm. The analytical flow was 1.0 mL/min and the protein concentrations of the samples were 1 mg protein/mL, with an injection volume of 0.5 mL.

Peptides identification

Peptides identification was performed by nano high-performance liquid chromatography coupled to an Orbitrap Elite mass spectrometer.[3]

1 mg of LPH was loaded on a 50 mg Bond Elut C18 EWP cartridge (Agilent, Santa Clara, USA). The cartridge was preliminary washed with 3 mL of acetonitrile (ACN) and conditioned with 3 mL of 0.1% trifluoroacetic acid (TFA); the sample was acidified with aqueous TFA to pH 2.5, then loaded, and the cartridge washed with 3 mL of 0.1% TFA. Peptides were eluted from the SPE column with 0.5 mL ACN/H_2O (50:50, v/v) containing 0.1% TFA, and were dried in a SpeedVac SC250 Express (Thermo Savant, Holbrook, NY, USA). The residue was reconstituted in 150 µL of 0.1% formic acid in H_2O for analysis.

The samples were separated on an EASY-Spray column (Thermo Fisher Scientific, 15 cm × 75 µm i.d. PepMap C18, 3 µm particles, 100 Å pore size) operated at 300 nL/min and 35 °C. Mobile phases were H_2O (solvent A) and ACN (solvent B), both with 0.1% formic acid. The following linear gradient was used: 1% B for 5 min; 1–5% B in 2 min; 5–35% B in 90 min; 35–90% B in 3 min; finally, the column was washed at 90% B for 10 min and then equilibrated at 1% B for 29 min.

Peptide spectra were acquired in the 300–2000 m/z range at 30,000 resolution (full width at half maximum, FWHM, at m/z 400) for the full scan. MS/MS spectra were acquired at 15,000 resolution (FWHM, at m/z 400) in top 10 data-dependent acquisition mode with the rejection of singly charged ions and unassigned charge states.

Table S1. Composition of experimental Western diet

**Ingredients**	**%**
**Fat (2.12 kcal/g)**	
Lard	20.68
Soybean oil	2.91
**Carbohydrate (1.65 kcal/g)**	
Sucrose	20.09
Maltodextrin	11.65
Dextrin	8.48
Powdered cellulose	5.82
**Protein (0.83 kcal/g)**	
Casein - vitamin tested	23.30
**Vitamin Mix**	1.16
**Mineral Mix**	1.16
**Food additives**	
Potassium citrate. Tribasic monohydrate	1.92
Calcium phosphate	1.51
Calcium carbonate	0.64
L-Cystine	0.35
Choline bitartrate	0.23
Food colouring (FD&C Red No.40)	0.05
**Total energy (4.60 kcal/g)**	
Fat (ether extract)	46.1
Carbohydrates	35.8
Protein	18.1

Ingredients percentage of the high-fat Western diet and energy provided from the different macromolecules.

Table S2. Sensitivity and inter/intra assay precision of the assays used in this study.

	Sensitivity	Precision	
		**Inter assay**	**Intra assay**
**Blood-lipid profile**			
Total cholesterol	9.7 mg/dL	1.20%	1.0%
HDL cholesterol	3.09 mg/dL	1.50%	0.52%
Triglycerides	8.85 mg/dL	1.90%	1.6%
**Antioxidant analysis**			
CAT	2 nmol/min/mL	9.9%	3.8%
SOD	0.044 U/mL	10.1%	9.6%
GR	0.1 nmol/min/mL	10.0%	8.0%
GPx	50 nmol/min/mL	7.2%	5.7%
TAC	8.54 CRE	7.0%	3.5%
OxLDL	0.188 ng/mL	12.0%	8%

CAT, catalase; GPx, glutathione peroxidase; GR, glutathione reductase; OxLDL, oxidized low-density lipoprotein; SOD, superoxide dismutase; TAC, total antioxidant capacity.

Table S3. Primers sequences and qPCR conditions

Target gene	Primer sequences	Ta (°C)
Cxcl1	Fwd 5´-AGC TCC CTT GGT TCA GAA AAT-3´<br>Rev 5´-AAA TAG GAC CCT CAA AAG AAA-3´	52
Selp	Fwd 5´-ACA ACG AGC CCA ACA ACA AGA-3´<br>Rev 5´-GGG TAG CAG GAG CAG GTA TAG-3´	55
Cd36	Fwd 5´-ATT CAT TTG TTC AAG TTG TGC-3´<br>Rev 5´-AAA AGG TGG AAA GGA GGC TGC-3´	52
Sr-a	Fwd 5´-GGC TGG AGG GAA GTT GTC AAT AC-3´<br>Rev 5´-TCT GCT GCA TCC CAC TGG TGA-3´	54
Inos	Fwd 5´-ACG GAC GAG ACG GAT AG-3´<br>Rev 5´-GGG CTT CAA GAT AGG GA-3´	55
Hprt	Fwd 5´-TGT TGG ATA TGC CCT TGA CTA-3´<br>Rev 5´-TGC GCT CAT CTT AGG CT-3´	52-55

Name of target gene, forward (Fwd) and reverse (Rev) sequences of the primers, and annealing temperatures (Ta) used to perform the qPCR. The hipoxanthine phosphoribosyl transferase (*hprt*) gene was used as housekeeping gene and the mRNA relative expression levels were calculated using the $2^{-\Delta\Delta Ct}$ method. Cd36, cluster of differentiation 36; Cxcl1, Chemokine (C-X-C motif) ligand 1; Inos, inducible nitric oxide synthase; Selp, P-selectin; Sr-a, class A macrophage scavenger receptor.

Table S4. Flow cytometry antibodies characteristics

Antibody	Fluorochrome	Clone	Manufacturer
Anti-CD4	AF-700	GK1.5	eBioscience (CA, USA)
Anti-CD11b	APC-Cy7	M1/70	BD (NJ, USA)
Anti-IFN-γ	FITC	XMG1.2	eBioscience

Name of antibody, conjugated fluorochrome, clone of antibody producer, and manufacturer. AF, alexa fluor; APC, allophycocyanin; CD4, cluster of differentiation 4; CD11b, cluster of differentiation 11b; FITC, fluorescein isothiocyanate; IFN-γ, interferon-γ.

Table S5. Amino acids composition of LPH.

Amino acids composition	LPH
Glu+Gln	24.47 ± 0.17
Arg	11.60 ± 0.06
Asp + Asn	10.27 ± 0.12
Leu	8.55 ± 0.05
Ser	5.97 ± 0.16
Phe	4.95 ± 0.01
Lys	4.92 ± 0.02
Gly	4.52 ± 0.06
Ile	4.45 ± 0.01
Tyr	4.42 ± 0.12
Thr	4.05 ± 0.02
Ala	3.89 ± 0.07
Val	3.47 ± 0.07
His	2.36 ± 0.01
Pro	0.75 ± 0.01
Cys	0.56 ± 0.19
Met	0.44 ± 0.15
Trp	0.35 ± 0.02

Data of amino acids composition are expressed in g amino acids/100 g amino acids ± standard deviation. LPH, lupin protein hydrolysate.

Table S6. Raw data of antioxidant parameters.

**Parameters**	**Control**	**LPH**	**Difference**	**p-value**
CAT (nmol/min/mL)	47.53 ± 3.52	61.24 ± 5.11	+31.86%	**0.035**
SOD (U/ mL)	0.58 ± 0.03	0.70 ± 0.04	+14.16%	**0.019**
GPx (nmol/min/mL)	255.60 ± 26.67	289.10 ± 33.75	+13.10%	0.597
GR (nmol/min/mL)	19.90 ± 0.90	21.42 ± 0.92	+4.01%	0.430
TAC (Copper reducing equivalents -CRE-)	95.01 ± 19.03	121.40 ± 30.34	+19.62%	**0.036**
oxLDL (ng/mL)	2.92 ± 0.49	1.95 ± 0.30	-26.58%	**0.019**

Raw data of each antioxidant assay for each experimental group ± standard error of the mean (n = 15). The calculated percentage of the difference between LPH group and the control group. $p < 0.05$ was considered statistically significant. CAT, catalase; SOD, superoxide dismutase, GPx, glutathione peroxidase; GR, glutathione reductase; TAC, total antioxidant capacity; oxLDL, oxidized low-density lipoprotein colesterol.

Figure S1. Representative standard curve of the antioxidative assays used in this study.

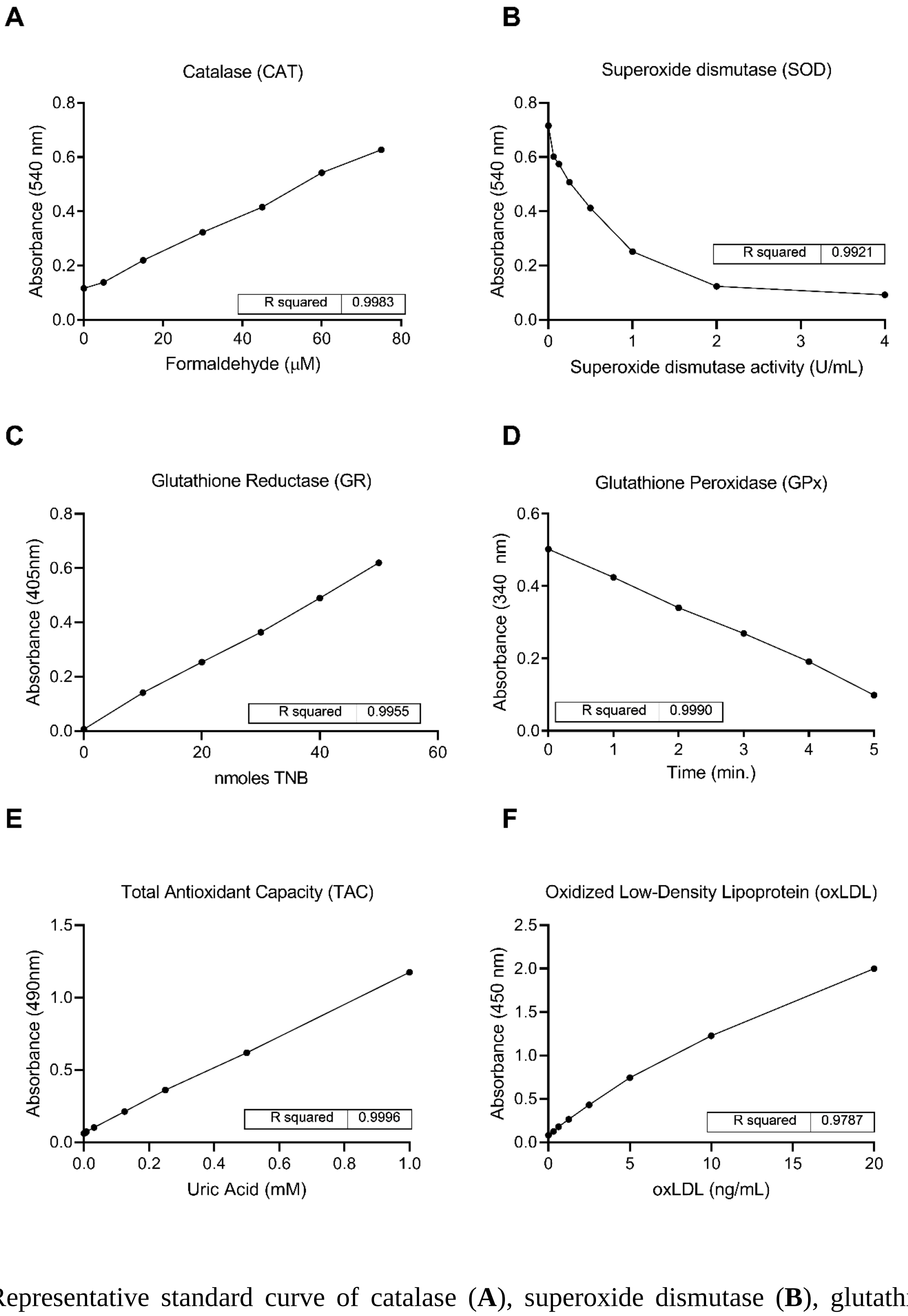

Representative standard curve of catalase (**A**), superoxide dismutase (**B**), glutathione reductase (**C**), glutathione peroxidase (**D**), total antioxidant capacity (**E**), and oxidized low-density lipoprotein (**F**).

Figure S2. Body weight monitored over time

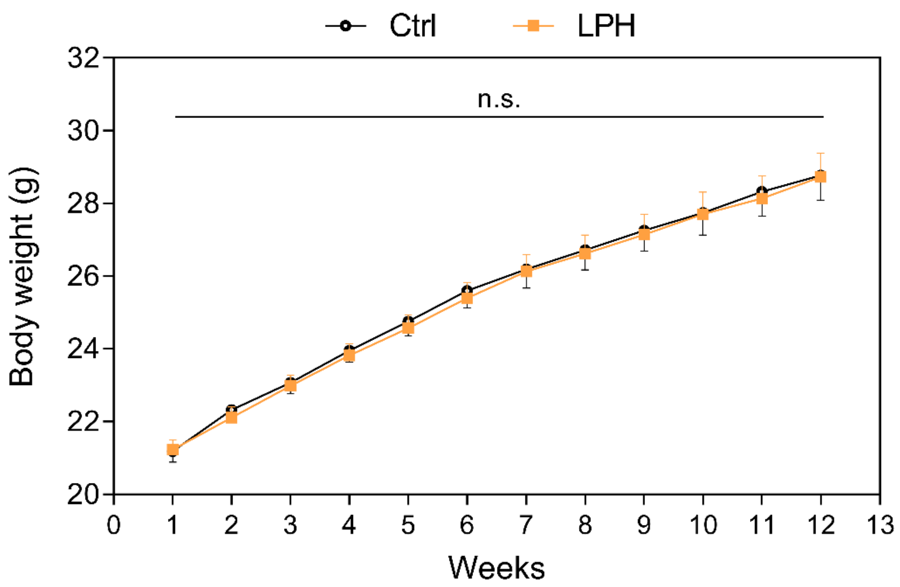

Effect of LPH on the evolution of body weight (*n* = 30). Results are expressed as as the mean and standard error of the mean of each group. Ctrl, control group; LPH, lupine protein hydrolysate group. n.s., not significant.

Figure S3. Cell proliferation and cell viability.

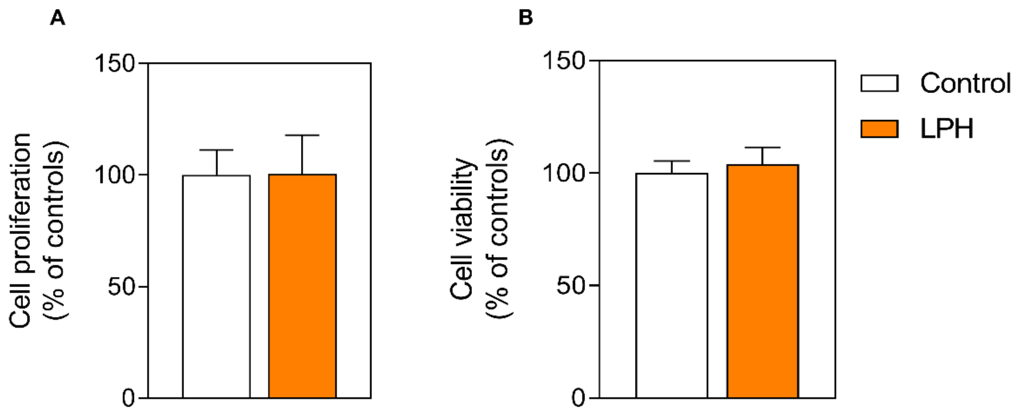

Cell proliferation in PHA-stimulated aortic cells of 12-weeks LPH-treated mice (**A**, *n* = 14). Cell viability in non-stimulated aortic cells of 12-weeks LPH-treated mice (**B**, *n* = 17). Results are expressed as the percentage of the control group and are shown as the mean and standard error of the mean of each group. LPH, lupine protein hydrolysate group; PHA, phytohaemagglutinin-P

References

1. Cruz-Chamorro, I.; Álvarez-Sánchez, N.; del Carmen Millán-Linares, M.; del Mar Yust, M.; Pedroche, J.; Millán, F.; Lardone, P. J.; Carrera-Sánchez, C.; Guerrero, J. M.; Carrillo-Vico, A., Lupine protein hydrolysates decrease the inflammatory response and improve the oxidative status in human peripheral lymphocytes. Food Res Int 2019, 126, 108585.
2. Adler-Nissen, J., Determination of the degree of hydrolysis of food protein hydrolysates by trinitrobenzenesulfonic acid. Journal of agricultural and food chemistry 1979, 27 (6), 1256-62.
3. Santos-Sánchez, G.; Cruz-Chamorro, I.; Bollati, C.; Bartolomei, M.; Pedroche, J.; Millan, F.; Millan-Linares, M. C.; Capriotti, A. L.; Cerrato, A.; Laganà, A.; Arnoldi, A.; Carrillo-Vico, A.; Lammi, C., A Lupinus angustifolius protein hydrolysate exerts hypocholesterolemic effect in western diet-fed-ApoE−/− mice through the modulation of LDLR and PCSK9 pathways. Food Function 2022, 13, 4158 - 4170.
